# Effect of polydopamine and fluoride ion coating on dental enamel remineralization: an in vitro study

**DOI:** 10.1186/s12903-023-03221-6

**Published:** 2023-07-26

**Authors:** Seok-Min Choi, Hee-Won Jung, Ji Hyun Ryu, Hyung-Keun You

**Affiliations:** 1 Department of Periodontology, Clinical Lab for Innovative Periodontology, School of Dentistry, Wonkwang University, Iksan, Jeonbuk 54538 South Korea; 2 Department of Carbon Convergence Engineering, Wonkwang University, Iksan, Jeonbuk 54538 South Korea; 3 Smart Convergence Materials Analysis Center, Wonkwang University, Iksan, Jeonbuk 54538 South Korea

**Keywords:** Dental caries, Tooth diseases, Tooth remineralization, Fluorides, Dental enamel

## Abstract

**Background:**

Fluoride treatment is one of the most effective dental caries prevention methods. To continuously prevent dental caries, stably immobilizing the fluoride on the tooth enamel is highly desirable. This study aimed to evaluate the remineralization of tooth enamels by one-pot coating using polydopamine and fluoride ions.

**Methods:**

To prepare the enamel specimens for polydopamine- and fluoride ion-coating, they were treated with polydopamine- and fluoride-containing gels. The enamel specimens were collected from human molars in a blind manner (*n* = 100) and were randomized into five treatment groups (*n* = 20, each): 1) untreated, 2) polydopamine-coated, 3) fluoride-containing gel-treated, 4) F varnish-treated, and 5) polydopamine- and fluoride ion-coated enamels. Vickers hardness number (VHN), morphology, and fluoride contents of the specimens were measured before and after the pH-cycling regimen.

**Results:**

Polydopamine- and fluoride ion-coated enamels showed the highest fluoride content and lowest VHN reduction among the samples. The fluoride content of the polydopamine/fluoride ion (PD/F)-coated enamel was increased to 182 ± 6.6%, which was far higher than that of the uncoated enamel (112.3 ± 32.8%, *P* < 0.05). The changes in the VHN values (ΔVHN) of PD/F-coated enamel substrates showed a slight reduction in the VHN (-3.6%, *P* < 0.05), which was far lower than that in the control group (-18.9%, *P* < 0.05). In addition, scanning electron microscopy clearly supported the effect of polydopamine- and fluoride ion-coatings on the remineralization of enamel specimens.

**Conclusion:**

Our findings suggest that one-pot treatments with polydopamine and fluoride ions could significantly enhance remineralization by inhibiting enamel demineralization through the prolonged retention of fluoride ions.

## Background

Oral diseases such as dental caries, endodontic disease, temporomandibular disorders, and periodontal disease can have systemic effects, increasing the host's susceptibility to other diseases [1–5]. Preventing these localized diseases may be a therapeutic solution for reducing the incidence of systemic diseases. The development of biomaterials and procedures to prevent dental caries has recently garnered growing interest. Dental caries is a common condition involving the demineralization of dental tissues by lactic and acetic acid produced from oral bacteria [6–10]. Fluoride treatments are highly effective in the prevention of dental caries in the clinic [11, 12]. Fluorides may interact with the minerals to form the dense crystal CaP structures resulting in the prevention of the demineralization [13–15]. Moreover, remineralization with fluoride treatments contributes to inhibiting the progression of dental caries and accelerating the formation of healthy minerals within the enamel in the early stages of caries formation [16, 17]. However, the effects of fluoride treatments are short-coming due to the lack of retention of fluorides on the enamel surface caused by the ceaseless changes in saliva and swallowing [18–23]. In addition, fluoride intake in certain doses causes acute toxicity leading to poisoning. The probable toxic dose of fluoride is reported to be 5 mg/kg of body weight [24–26]. Thus, even a small concentration may cause medical problems if frequently applied to prevent tooth decay. Although fluoride treatment with low concentrations inhibits demineralization [27], fluoride concentrations should be sustained in the oral cavity to prevent dental caries. Thus, it is highly desirable to stably immobilize fluoride on the tooth enamel to prevent dental caries.

Polydopamine (PD) is a simple and powerful surface coating material used for versatile applications in the environmental, energy, and biomedical fields [28–30]. In general, PD can be applied on various surfaces, including polymers, metals, and ceramics such as hydroxyapatites, by the co-contributions of covalent polymerization and non-covalent self-assembly [28–30]. Furthermore, one-pot coating of PD with molecules (i.e., polymers, proteins, peptides, and ions) allows the co-immobilization of PD and target molecules by a simple auto-oxidation approach of PD [29, 30]. In particular, PD coating is biocompatible and can be used in various biomedical applications including dental biomedical devices [29, 30]. For instance, PD-coated titanium implant materials exhibit excellent corrosion resistance compared with that of uncoated titanium [31]. Furthermore, one-pot dopamine-mediated biomineralizations of calcium carbonate are achieved during PD nanoparticle formation [32]. Thus, the PD coating method is one of the significant candidate methods for the immobilization of fluoride ions.

We hypothesized that the co-immobilization of PD and fluoride can prolong the retention of fluoride on the enamel surface, resulting in the successful prevention of dental caries by inhibiting demineralization. The study aimed to evaluate the remineralization effects of one-pot PD/F treatments on enamel substrates in vitro.

## Methods

### Preparation of enamel specimens

This study was approved by our Institutional Review Board of Wonkwang University Dental Hospital (approval number: WKDIRB 201903–03). All procedures performed in this study involving human teeth were conducted with written informed consent in accordance with the Declaration of Helsinki. Enamel specimens were obtained from patients with periodontal disease at the Department of Periodontology of Wonkwang University Dental Hospital. Enamel specimens from patients with fluorosis and amelogenesis imperfecta were excluded. Among the extracted teeth, only those with no lesions such as caries, abrasion, and corrosion in the crown, were selected. The teeth were cut using a cutting disc (0.3 mm, Sejong Dental, Seoul, Korea). The enamel specimens (3 × 3 × 0.5 mm) were positioned with their labial aspect parallel to a slide glass and molded using acrylic resin (Cold-curing acrylic, Vertex-Dental B.V., Zeist, Netherlands). The molded enamel specimens were stored at 18–20 °C and 100% relative humidity before the initial surface microhardness measurements.

### Preparation of PD/F-coated enamel specimens

To prepare the enamels for PD/F coating, we collected specimens (*n* = 150) from patients and measured their Vickers hardness numbers (VHNs). The enamel specimens with Vickers hardness numbers (VHNs) 300–400 [33] were selected (*n* = 100) and randomized into five treatment groups (*n* = 20/group): 1) Con (negative control), the specimens were rinsed with distilled water; 2) PD, the specimens were coated with a dopamine solution (50 mM); 3) NaF, the specimens were treated with a 2% NaF gel (Natural-F Gel, Denbio, Korea); 4) Var, the specimens were treated with 5% sodium fluoride varnish (Enamelast, 0.4 ml; Ultradent, South Jordan, UT, USA); and 5) PD/F, the specimens were coated with a mixture of PD (50 mM) and 2% NaF. To prepare the PD-coated enamel specimens, dopamine hydrochloride (Sigma-Aldrich, USA) was dissolved in Tris–HCl buffered solution at a molar concentration of 50 mM. Enamel specimens were treated with dopamine solution (50 mM) using a dental micro-brush. For the NaF- and Var-treated enamel specimens, both were applied to enamels using a micro-brush, respectively. To prepare the PD/F-coated enamels, dopamine solution (50 mM) was mixed with the 2% NaF gels at a volume ratio of 2:1. The mixture solutions of dopamine and NaF gels were applied onto the enamel specimens using a micro-brush. All samples were rinsed with distilled water after 1 h incubation at 18–20 °C before being subjected to a pH-cycling regimen.

### pH-cycling experiments

To validate the caries control effect of PD/F coating on the enamel in vitro, we used a previously reported pH-cycling model [34, 35]. Although the model has several limitations with simplified artificial intraoral conditions, it is suitable for monitoring the efficacy of remineralization under similar caries formation conditions involving the loss and gain of minerals [34, 36]. The enamel specimens were subjected to pH cycling for 5 days. The pH cycling procedure consisted of demineralizing solution (2.0 mM calcium, 2.0 mM phosphate, 75 mM acetate buffer, and 0.04 µg fluoride/mL; pH 4.7) for 8 h and remineralizing solution (1.5 mM calcium, 0.9 mM phosphate, 150 mM KCl, 100 mM cacodylic buffer, and 0.05 mg fluoride/mL; pH 7.0) for 16 h once daily. All specimens were washed with distilled deionized water (DDW) after each step. During pH cycling, the demineralizing and remineralizing solutions were replaced on days 2 and 4.

### Characterization of PD/F-coated enamel substrates

The morphologies of the PD/F-coated enamel specimens were analyzed using scanning electron microscopy (SEM, S-4800, Hitachi Ltd., Tokyo, Japan) with an acceleration voltage of 15 kV at the Core Facility for Supporting Analysis & Imaging of Biomedical Materials in Wonkwang University and was supported by the National Research Facilities and Equipment Center. The specimens were coated with platinum before SEM imaging. Additionally, the surface elemental compositions of the specimens were investigated using X-ray photoelectron spectroscopy (XPS, K-Alpha, Thermo Fisher Scientific, Loughborough, UK). The differences in the fluoride contents of the surfaces were evaluated by comparing the final fluoride content (at%) after treatment with the mineralization solution with the initial fluoride content (at%). The difference in the fluoride content (x) was calculated using the following equation:

$$\mathrm{x }\left(\mathrm{\%}\right)=\frac{final\;fluoride\;content \left(at\%\right)-initial\;fluoride\;content \left(at\%\right)}{initial\;fluoride\;content \left(at\%\right)}\times 100(\mathrm{\%})$$

### Measurement of surface microhardness

The surface microhardness of the samples was measured using a microhardness tester (MXT70, Matsuzawa Co., Ltd., Akita, Japan) with a 100-g load, applied for 15 s. A microhardness tester was used as described below to minimize errors. Briefly, the VHN of each specimen was measured four times, and indentations were made at intervals of more than 50 µm. Additionally, the values corresponding to the two indents on the edges were ignored and the averages of the values corresponding to the two remaining indents were considered. Finally, computer monitor and camera (IMTcam3, P/N: TP603100A, i-Solution Inc., Penang, Malaysia) were used to image the indents and ensure that the indent borders lay within the inner surfaces of the indentation bars. To measure microhardness, we used the Vickers method with VHN. VHN is generally useful in confirming the differences in physical properties after chemical/physical treatments of enamel substrates [37, 38]. For instance, monitoring VHN changes before/after pH-cycling is a simple method to evaluate enamel remineralization [39, 40]. The changes in microhardness were evaluated by comparing the VHN values after treatment with the mineralization solution and the initial VHN values. The change in the microhardness (y) was calculated using the following equation:

$$\mathrm{y }\left(\mathrm{\%}\right)=\frac{{VHN}_{Day5}- {VHN}_{initial}}{{VHN}_{initial}}\times 100 (\mathrm{\%})$$

### Statistical analysis

All statistical analyses were performed using SPSS, version 12.0 (SPSS Inc., Chicago, IL, USA). Statistical significance was analyzed using Student’s t-test for two groups and one-way analysis of variance for multi-group comparisons. To test data normality, we used both the Kolmogorov–Smirnov and Shapiro—Wilk methods with the Q-Q plots. The results are presented as mean ± SEM. *P* < 0.05 was considered statistically significant. All experiments were performed at least three times.

## Results

### Formation of PD/F-coated enamel substrates

**Fig. 1:**
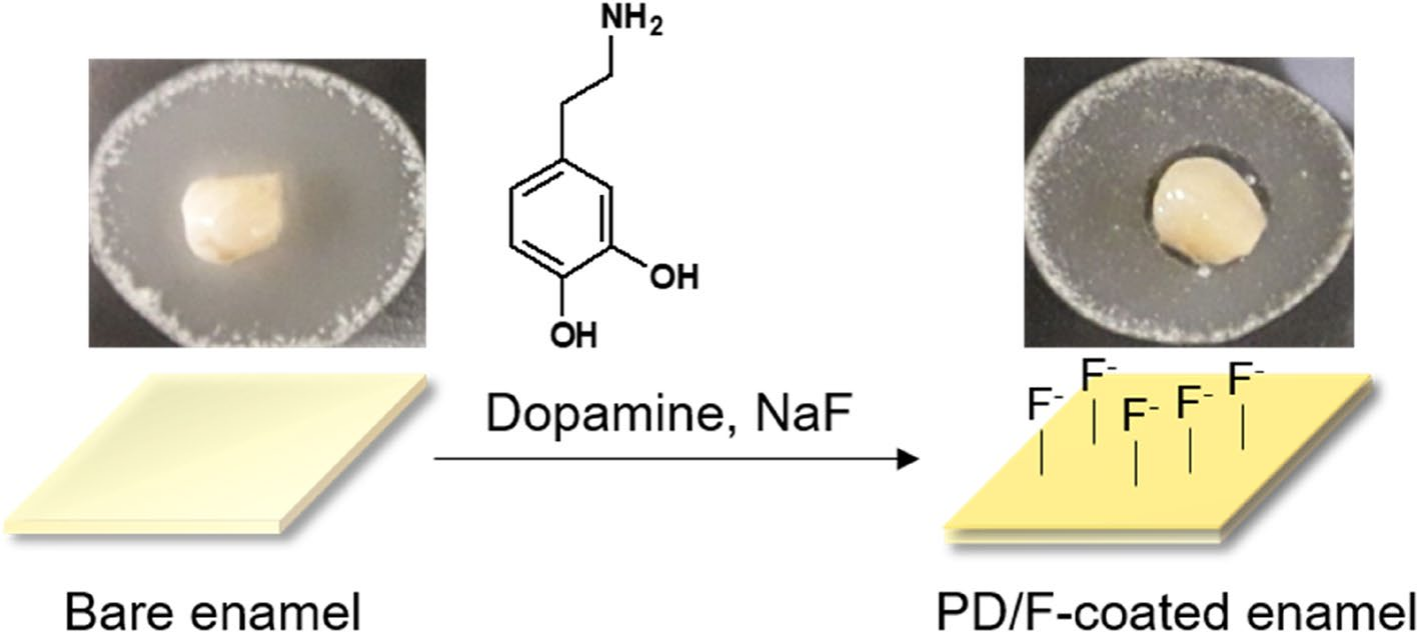
Schema and photographic images of PD/F-coated enamel substrates

**Fig. 2:**
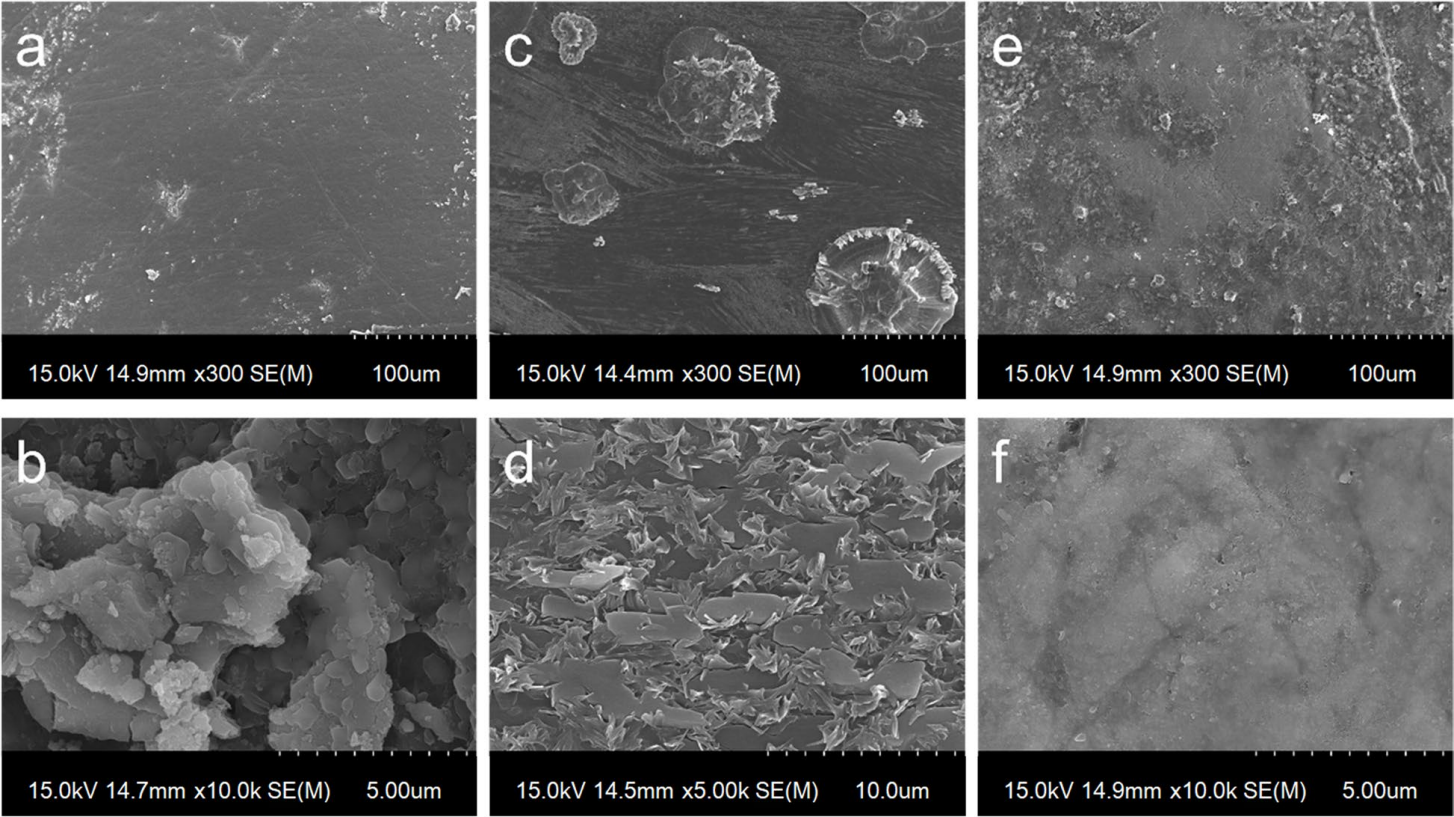
SEM images of Con (**a**, **b**), NaF-treated (**c**, **d**), and PD/F-coated enamel (**e**, **f**) with × 300 and × 10,000 magnifications

**Fig. 3:**
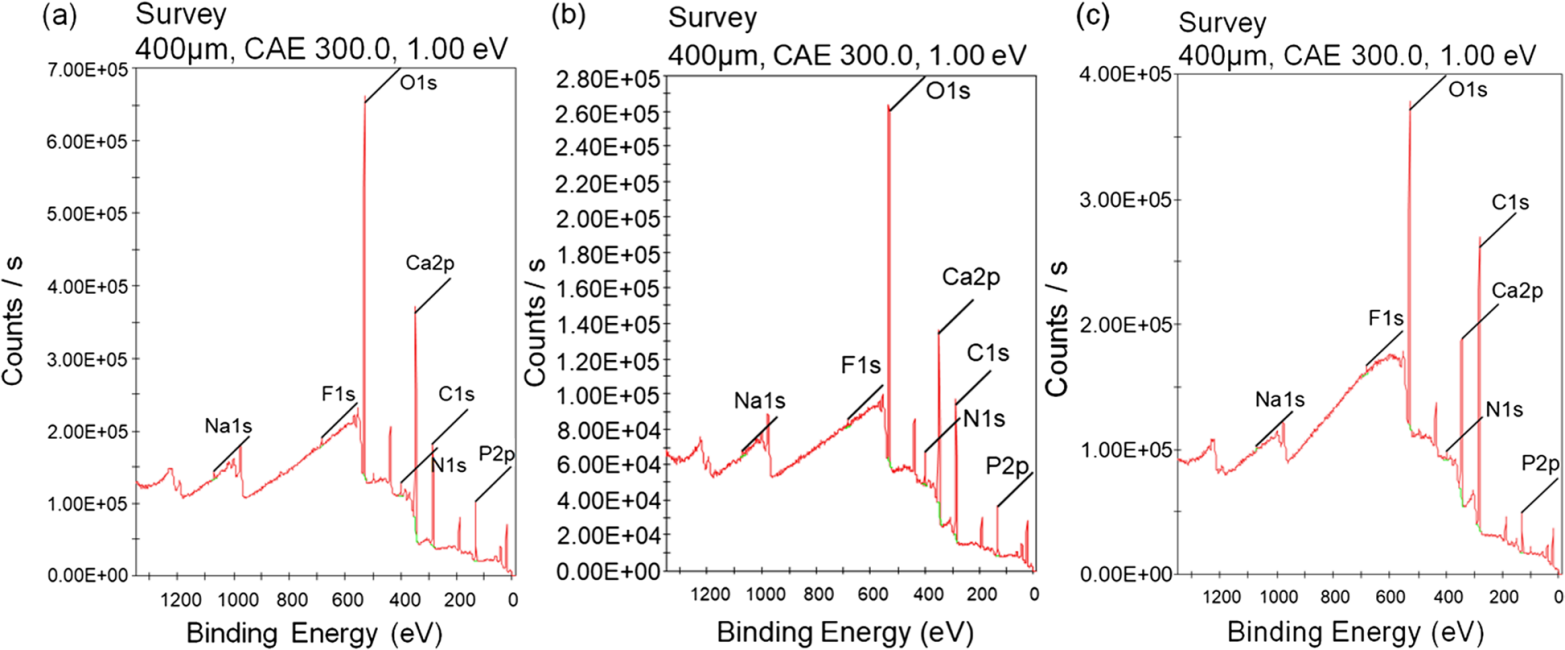
XPS spectra of (**a**) Con, (**b**) NaF-treated, and (**c**) PD/F-coated enamels

To prepare the PD/F-coated enamel substrates, the enamel surfaces were vigorously washed with DDW, and the dopamine (50 mM)-containing 2% NaF gels were treated with the enamel substrates (Fig. 1). As shown in Fig. 1, the photographs of the enamel specimens did not significantly differ before and after PD/F coating. To confirm the PD/F coating on the enamel, we monitored morphological changes in the specimens. Compared with that of the unmodified enamel substrates (Fig. 2a, b), the PD/F-coated enamel (Fig. 2c, d) showed a reduction in surface roughness, indicating that the specimens were coated with PD/F. The NaF gel-treated enamel exhibited similar morphological changes (Fig. 2e, f). However, heterogeneous morphological changes were observed during drying (Fig. 2c). Moreover, multiple cracks on the enamel surfaces were observed in the SEM images of the NaF gel-treated enamel with a × 10,000 magnification (Fig. 2d). This was probably because of the polymeric components of the NaF gels during the drying steps. Furthermore, Ca_2p_/C_1S_ ratio (0.16) of the PD/F-coated enamels in the XPS spectra was lower than those of uncoated (0.60) and NaF-coated enamels (0.30) (Fig. 3).

### Remineralization of PD/F-coated enamel substrates

**Fig. 4:**
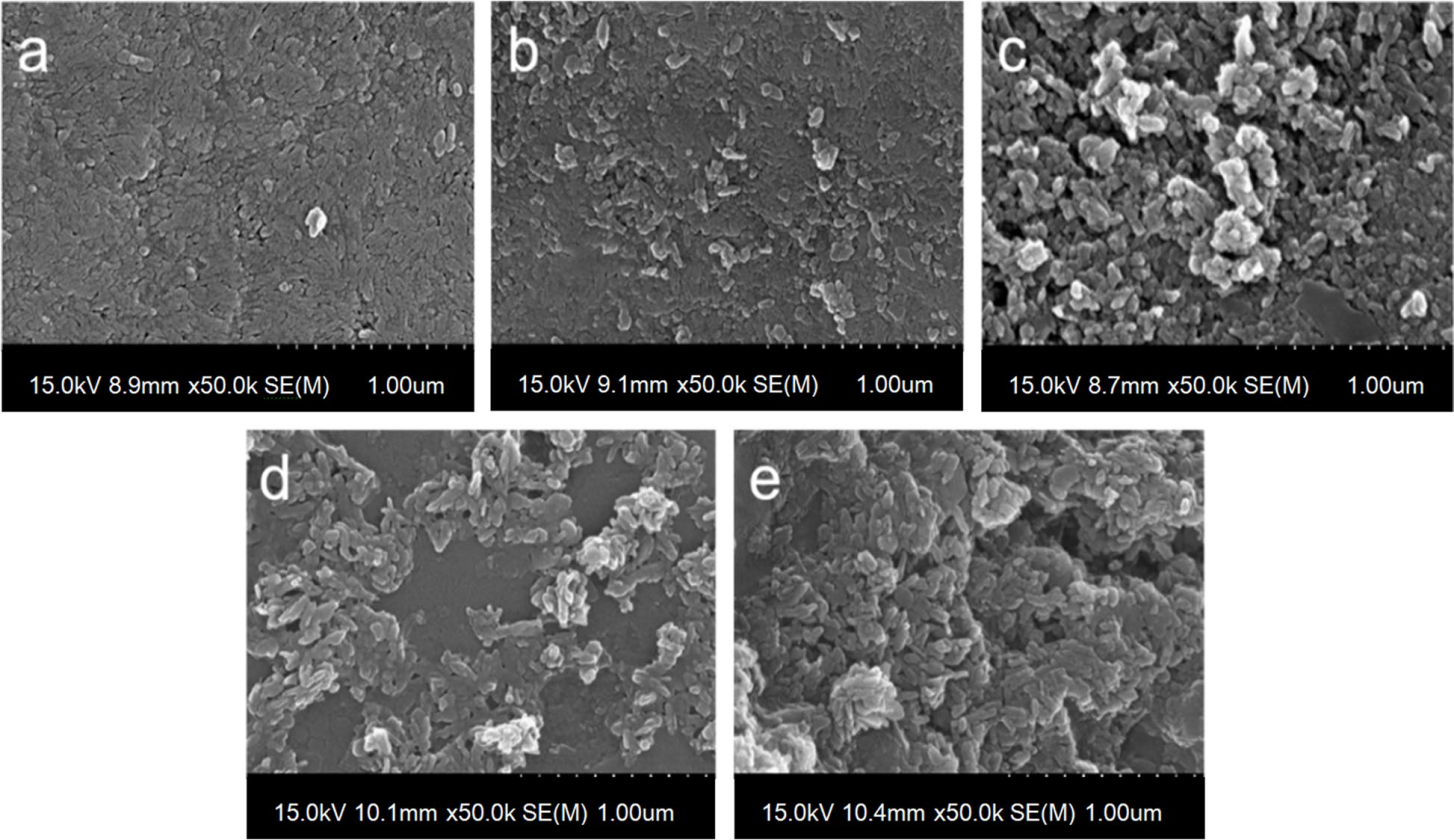
SEM images of (**a**) Con, (**b**) PD-coated, (**c**) NaF-coated, (**d**) Var-coated, and (**e**) PD/F-coated enamel specimens after remineralization

We evaluated the remineralization effects of the PD/F-coated enamel using morphological changes and elemental composition measurements. As shown in Fig. 4, the morphologies of the PD/F-coated enamel surfaces clearly exhibited remineralization effects after the pH cycling experiments. Unlike the bare enamel surfaces (Fig. 4a), CaP clusters were found on the PD, NaF, and Var-coated enamel surfaces (Fig. 4b, c, and d). For the PD/F-coated enamel surfaces, the CaP clusters were significantly larger than those of the other specimens. In addition to the morphological analysis, the changes in the fluoride content and Ca/P ratio supported the remineralization of PD/F-coated enamel. The fluoride content of the PD/F-coated enamel had changed to 182.0 ± 6.6%, which was higher than those of the uncoated (112.3 ± 32.8%), PD-coated (71.7 ± 13.8%), NaF-coated (107.5 ± 13.8%), and Var-coated (134.9 ± 25.3%) enamel surfaces (*P* < 0.05). In addition, differences in the F-contents between Con and NaF-coated groups were not significant (*P* > 0.05) that might be due to the instability of fluoride immobilization on the enamels during the pH cycling experiments. Moreover, the Ca-P ratios of all samples measured by XPS were between 1.28 to 1.36, as listed in Table 1.

**Table 1:** F content changes and Ca/P ratios

Group	Difference in F content	Ca-to-P ratio (Ca/P)
Con	112.3 ± 32.8^*^	1.285
PD	71.7 ± 13.8^*^	1.292
NaF	107.5 ± 4.9^*^	1.346
Var	135.0 ± 25.3^*^	1.338
PD/F	182.0 ± 6.6^+^	1.355

$$\mathrm{Difference\;in\;F\;content}= \frac{final\;fluoride\;content \left(at\mathrm{\%}\right)-initial\;fluoride\;content \left(at\mathrm{\%}\right)}{initial\;fluoride\;content \left(at\mathrm{\%}\right)}\times 100(\mathrm{\%})$$

^+^Indicates a significant difference between changes in F contents compared to Con (*P* < 0.05)

^*^Indicates a significant difference between changes in F contents compared to PD/F (*P* < 0.05)

### Surface microhardness of PD/F-coated enamel substrates

**Fig. 5:**
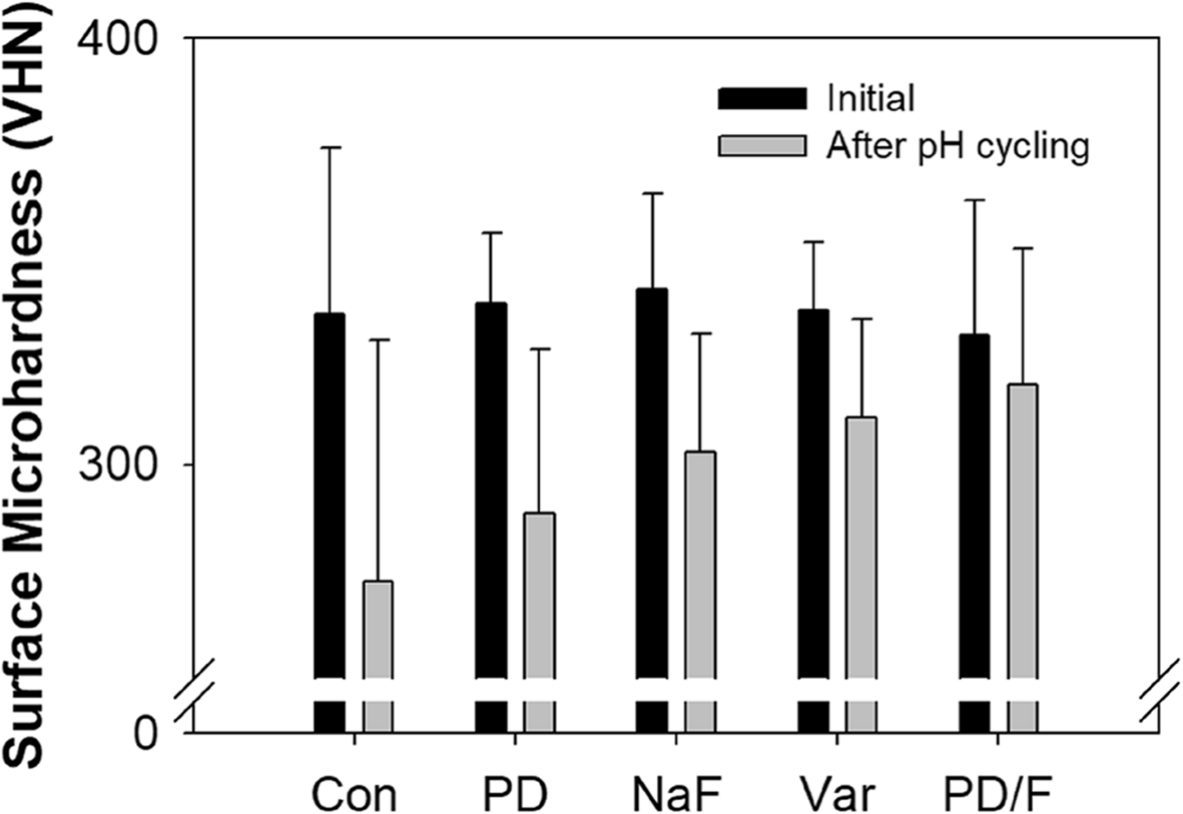
Differences in initial surface microhardness values and those measured after pH cycling for 5 days

Next, the surface microhardness of the PD/F-coated enamel was measured using a microhardness tester. In general, microhardness is among the most important indexes of the degree of remineralization [26–28]. The VHNs of enamel specimens before pH cycling were between 300 and 400 with a p value of 0.793, indicating no significant differences in the initial microhardness among the control and experimental groups (Fig. 5). The microhardness after the pH cycling regimen was decreased by the demineralization of the enamel specimens. The changes in VHN values (ΔVHN) of PD/F-coated enamel substrates showed a slight reduction in VHNs (-3.6%) compared with any material applied (-18.9%), PD-coated (-14.7%), NaF-coated (-11.1%), and Var-coated (-7.4%) enamel species (Fig. 5 and Table 2). The fluoride-treated groups, including PD/F-coated, NaF-coated, and Var-coated groups, showed significantly enhanced surface microhardness compared with those of control groups (*P* < 0.05). Furthermore, no significant differences were observed between Con (-18.93) and PD (-14.73, *P* = 0.227), or between NaF (-11.07) and Var (-7.44, *P* = 0.068).

**Table 2:** Surface microhardness values

Group	VHN_initial_ (Mean ± SD)	VHN_Day 5_ (Mean ± SD)	ΔVHN Mean
Con	335.10 ± 39.11	272.61 ± 56.58	-18.93
PD	337.71 ± 16.49	288.56 ± 38.33	-14.73
NaF	340.90 ± 22.53	303.07 ± 27.64	-11.07^*^
Var	336.14 ± 15.90	310.93 ± 23.10	- 7.44^*^
PD/F	330.34 ± 31.54	318.45 ± 32.13	- 3.61^*^

^*^Indicates a significant difference between changes in VHN compared to Con (*P* < 0.05)

$$\Delta \mathrm{VHN }=\frac{{VHN}_{Day 5}- {VHN}_{initial}}{{VHN}_{initial}}\times 100 (\mathrm{\%})$$

## Discussion

**Fig. 6:**
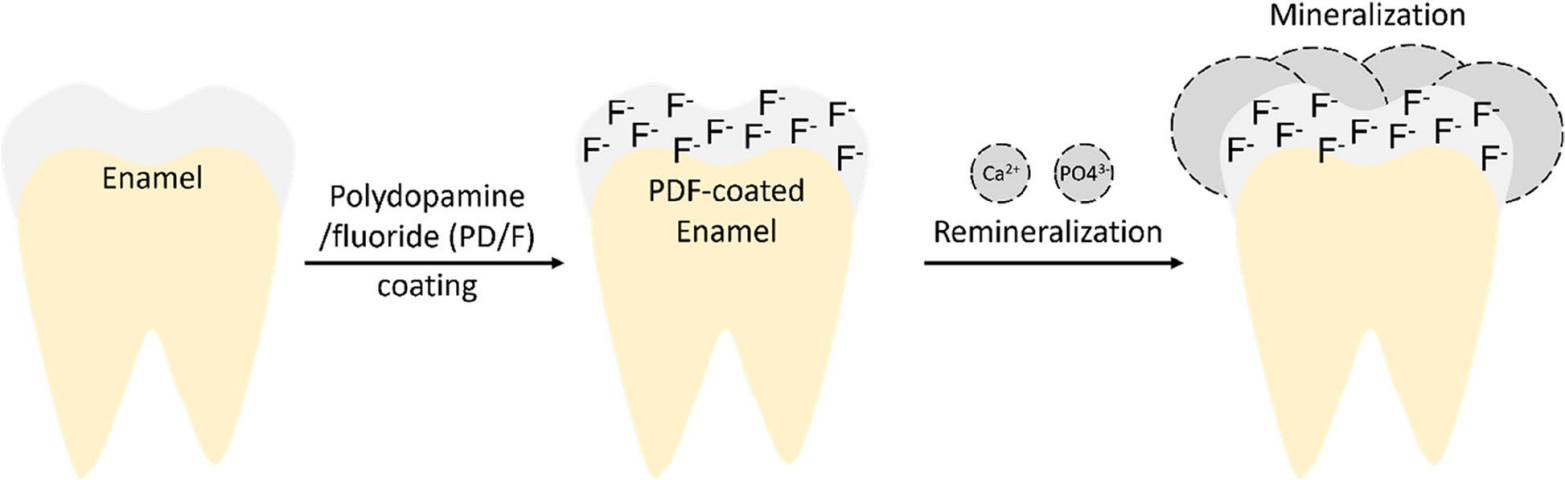
Schematic representation of tooth remineralization by forming a PD/F hybrid coating layer on tooth enamel

Dental caries is highly preventable, yet it remains common [41, 42]. The development of carious lesions is restricted by using sugar alcohols (i.e., xylitol and erythritol as substitutes for sugar), pit and fissure sealants, and topical fluoride application [43–45]. In particular, the application of fluoride in a dental clinic is a notably effective and inexpensive method for caries prevention by controlling fluoride content and/or the use of additives [11, 23, 46–50]. For the long-lasting retention of fluoride ions on the enamel, we used PD-based one-pot coating methods. One-pot PD coating with polymers, biomolecules, and ions can be co-immobilized on the substrates by preparing a coating solution with dopamine and molecules [29]. As shown in Fig. 6, the PD/F-coated enamel are expected to remineralize on the surfaces, resulting in the prevention of dental caries.

Morphological and elemental composition studies using SEM and XPS clearly revealed the effectiveness of PD/F coating on enamel in preventing demineralization. The CaP clusters on the PD/F-coated enamel substrates were larger than those on other species, including PD-coated enamel and F-containing material treatments (NaF, Var). Moreover, the differences in F content before and after pH-cycling experiments with demineralization and remineralization clearly showed that PD/F coating on the enamel significantly enhanced the retention of fluorides on the surfaces. The F content difference in PD/F-coated enamel specimens after pH-cycling was 182.0 ± 6.6%, which was higher than those in the uncoated (112.3 ± 32.8%), PD-coated (71.7 ± 13.8%), NaF-coated (107.5 ± 4.9%), and Var-coated (135.0 ± 25.3%) enamel specimens. In addition, our data suggests that PD coating alone does not provide significant changes, but PD/F coating is effective in enamel remineralization. PD can bind with Ca^2+^ ions resulting in the nucleation of CaP crystals [30, 51] and fluorides significantly affect bone/tooth formation, microstructure, mineralization, and microhardness [52, 53]. Although coating the enamel alone with PD has no significant effect on its remineralization, PD coating the dentin significantly enhanced its remineralization [54], consistent with our findings regarding PD/F-coated enamel specimens.

The surface microhardness of enamel directly correlates with its mineral composition, and microhardness can be used to evaluate the effects of remineralization treatments [14, 55, 56]. Monitoring microhardness using VHNs before and after pH cycling is a simple method to confirm the effects of the physical/chemical treatment on enamel substrates [33, 41, 42]. Moreover, using the pH cycling test to replicate the conditions within the mouth for 5 to 7 days is suitable for evaluating remineralization [23, 57, 58]. Preliminary pH cycling was performed for 7 days under the same conditions as those used for the experiments, with no significant differences between 5 and 7 days. PD/F coating of the enamel showed a slight reduction in VHNs (-3.6%), which was far lower than those of any material applied (-18.9%), PD-coated (-14.7%), NaF-coated (-11.1%), and Var-coated (-7.4%) enamel specimens, indicating that successful remineralizations of enamels were achieved. Considering fluoride-containing materials, the PD/F coating methods prolonged the immobilization of fluorides with PD.

Regarding clinical applications, there are concerns on the detachment of PD and fluoride ions caused by saliva and swallowing. As aforementioned, fluoride intake can potentially cause acute toxicity, and the fluoride content of the PD/F-coated enamel increased to 55.4% after pH cycling. In addition, we used commercially available fluoride gels (2% NaF) with the non-toxic amount of 0.05 mL/sample, and conventional fluoride treatment for clinical use. Generally, the incubation time for PD coatings is 5–6 h in Tris pH 8.5 [29]. However, we applied PD/F with a cotton swab suitable for clinical applications, which requires a few seconds. Thus, PD/F coating can be used in various dental applications, including drug delivery, tissue engineering, and as a biomedical device in surgical procedures.

Our study was limited by experimental conditions. Although a pH-cycling model was used to simulate the conditions within the mouth, various factors associated with remineralization, such as the flow of the oral fluids (saliva and biofilm fluid) and their compositions, have not been replicated [16, 59]. Therefore, further studies are needed to investigate the application of PD/F coating methods to oral environments to improve their accuracy. In addition, we only focused on microhardness for measuring the in vitro remineralization effects of PD/F treatments on the enamel. Further studies are required to evaluate remineralization using various techniques (such as, microradiography, iodide and urea permeability, and mineral content measuring). The black color of PD would be an aesthetic consideration in actual clinical use. Additionally, studies assessing PD/F coating enamels in animal models or preclinical trials should be performed. In addition to the in vivo assessments, long-term toxicity should be validated before the clinical use of PD/F treatments in dental caries prevention.

## Conclusions

This study aimed to evaluate the remineralization effects of one-pot PD/F treatments on enamel substrates. PD/F coating the enamel immobilized the fluoride ions with PD, facilitating fluoride retention. The SEM images and XPS spectra demonstrated the fluoride immobilization on the substrates. In addition, the CaP clusters in the PD/F-coated enamels after pH-cycling were significantly larger than those in the other specimens. Moreover, compared to untreated enamels, PD/F-coated enamels enhanced surface microhardness. Our study found that PD/F-coating is more effective at remineralizing enamel than conventional fluoride treatment methods. This suggests that one-pot coating of PD and fluorides could improve the efficacy of fluoride treatment in clinical dentistry. Thus, PD/F-coated biomedical devices and one-pot PD/F coating methods can be exploited for various dental applications, including tooth enamel remineralization.

## Data Availability

All data generated or analysed during this study are included in this published article. Data supporting this research article are available from the corresponding authors on reasonable request.

## Abbreviations

*VHN*: Vickers hardness number
*PD*: Polydopamine
*F*: Fluoride ion
*NaF*: Fluoride-containing gel
*Var*: Varnish
*DDW*: Distilled deionized water
*SEM*: Scanning electron microscopy
*XPS*: X-ray photoelectron spectroscopy

## References

[1] Scannapieco FA (2013). The oral microbiome: its role in health and in oral and systemic infections. Clin Microbiol Newsl.

[2] Le X, Kolltveit KM, Tronstad L, Olsen I (2020). Systemic diseases caused by oral infection. Clin Microbiol Rev.

[3] Niazi SA, Bakhsh A (2022). Association between endodontic infection, its treatment and systemic health: a narrative review. Medicina.

[4] Minervini G, Franco R, Marrapodi MM, Fiorillo L, Cervino G, Cicciu M (2023). Prevalence of temporomandibular disorders in children and adolescents evaluated with diagnostic criteria for temporomandibular disorders: a systematic review with meta-analysis. J Oral Rehabil.

[5] Minervini G, Franco R, Marrapodi MM, Mehta V, Fiorillo L, Badnjevic A (2023). The association between COVID-19 related anxiety, stress, depression, temporomandibular disorders, and headaches from childhood to adulthood: a systematic review. Brain Sci.

[6] Warreth A. Dental caries and its management. Int J Dent. 2023;2023:9365845. 10.1155/2023/9365845 PMC9831703 36636170

[7] Cheng L, Zhang L, Yue L, Ling J, Fan M, Yang D (2022). Expert consensus on dental caries management. Int J Oral Sci.

[8] Kiros A, Saravanan M, Niguse S, Gebregziabher D, Kahsay G, Dhandapani R, et al. Bacterial profile, antimicrobial susceptibility pattern, and associated factors among dental caries-suspected patients attending the Ayder comprehensive specialized hospital and private dental clinic in Mekelle, northern Ethiopia. BioMed Res Int. 2022;2022:3463472. 10.1155/2022/3463472 PMC9592214 36299705

[9] Selwitz RH, Ismail AI, Pitts NB (2007). Dental caries. Lancet.

[10] Kidd E (2011). The implications of the new paradigm of dental caries. J Dent.

[11] Petersen PE, Lennon MA (2004). Effective use of fluorides for the prevention of dental caries in the 21st century: the WHO approach. Community Dent Oral Epidemiol.

[12] Whelton HP, Spencer AJ, Do LG, Rugg-Gunn AJ (2019). Fluoride revolution and dental caries: evolution of policies for global use. J Dent Res.

[13] Everett ET (2011). Fluoride’s effects on the formation of teeth and bones, and the influence of genetics. J Dent Res.

[14] Attin T, Kielbassa AM, Schwanenberg M, Hellwig E (1997). Effect of fluoride treatment on remineralization of bleached enamel. J Oral Rehabil.

[15] Lussi A, Hellwig E, Ganss C, Jaeggi T (2009). Buonocore Memorial Lecture. Dental erosion Oper Dent.

[16] Cury JA, Tenuta LMA (2009). Enamel remineralization: controlling the caries disease or treating early caries lesions?. Braz Oral Res.

[17] Gangrade A, Gade V, Patil S, Gade J, Chandhok D, Thakur D (2016). In vitro evaluation of remineralization efficacy of different calcium- and fluoride-based delivery systems on artificially demineralized enamel surface. J Conserv Dent.

[18] Margalit D, Gedalia I (1969). Release of fluoride into saliva after topical fluoride application. J Dent Res.

[19] Lincir I, Rosin-Grget K (1993). Caries-preventive effect of two different topical fluoride concentrations with two different frequencies of application in preschool children. Caries Res.

[20] Ogard B, Seppä L, Rølla G (1994). Professional topical fluoride applications–clinical efficacy and mechanism of action. Adv Dent Res.

[21] Seppä L (1988). Effects of a sodium fluoride solution and a varnish with different fluoride concentrations on enamel remineralization in vitro. Scand J Dent Res.

[22] Nammour S, Demortier G, Florio P, Delhaye Y, Pireaux JJ, Morciaux Y (2003). Increase of enamel fluoride retention by low fluence argon laser in vivo. Lasers Surg Med.

[23] Magalhães AC, Comar LP, Rios D, Delbem AC, Buzalaf MA (2008). Effect of a 4% titanium tetrafluoride (TiF4) varnish on demineralisation and remineralisation of bovine enamel in vitro. J Dent.

[24] Ullah R, Zafar MS, Shahani N (2017). Potential fluoride toxicity from oral medicaments: a review. Iran J Basic Med Sci.

[25] Bayless JM, Tinanoff N (1985). Diagnosis and treatment of acute fluoride toxicity. J Am Dent Assoc.

[26] Shulman JD, Wells LM (1997). Acute fluoride toxicity from ingesting home-use dental products in children, birth to 6 years of age. J Public Health Dent.

[27] Buzalaf MAR, Pessan JP, Honório HM (2011). Mechanisms of action of fluoride for caries control”. Monogr Oral Sci.

[28] Lee H, Dellatore SM, Miller WM, Messersmith PB (2007). Mussel-inspired surface chemistry for multifunctional coatings. Science.

[29] Ryu JH, Messersmith PB, Lee H (2018). ’Polydopamine surface chemistry: A decade of discovery’. ACS Appl Materilas Interfaces.

[30] Lee HA, Ma Y, Zhou F, Hong S, Lee H (2019). Material-independent surface chemistry beyond polydopamine coating. Acc Chem Res.

[31] Lee JJ, Park IS, Shin GS, Lyu S, Ahn S, Bae T (2014). Effects of polydopamine coating on the bioactivity of titanium for dental implants. Int J Precis Eng Manuf.

[32] Dong Z, Feng L, Hao Y, Chen M, Gao M, Chao Y (2018). Synthesis of hollow biomineralized CaCO3-polydopamine nanoparticles for multimodal imaging-guided cancer photodynamic therapy with reduced skin photosensitivity. J Am Chem Soc.

[33] Zantner C, Beheim-Schwarzbach N, Neumann K, Kielbassa AM (2007). Surface microhardness of enamel after different home bleaching procedures. Dent Mater.

[34] Buzalaf MA, Hannas AR, Magalhães AC, Rios D, Honório HM, Delbem AC (2010). pH-cycling models for in vitro evaluation of the efficacy of fluoridated dentifrices for caries control: strengths and limitations. J Appl Oral Sci.

[35] Queiroz CS, Hara AT, Paes Leme AF, Cury JA (2008). pH-cycling models to evaluate the effect of low fluoride dentifrice on enamel de- and remineralization. Braz Dent J.

[36] Amaechi BT (2019). Protocols to study dental caries in vitro: pH cycling models. Methods Mol Biol.

[37] Jabbarifar SE, Salavati S, Akhavan A, Khosravi K, Tavakoli N, Nilchian F (2011). Effect of fluoridated dentifrices on surface microhardness of the enamel of deciduous teeth. Dent Res J (Isfahan).

[38] Chuenarrom C, Benjakul P, Daosodsai P (2009). Effect of indentation load and time on knoop and Vickers microhardness tests for enamel and dentin. Mater Res.

[39] Bhadoria N, Gunwal MK, Kukreja R, Maran S, Devendrappa SN, Singla S (2020). An in vitro evaluation of remineralization potential of functionalized tricalcium phosphate paste and CPP-ACPF on artificial white spot lesion in primary and permanent enamel. Int J Clin Pediatr Dent.

[40] Yesilyurt C, Sezer U, Ayar MK, Alp CK, Tasdemir T (2013). The effect of a new calcium-based agent, Pro-Argin, on the microhardness of bleached enamel surface. Aust Dent J.

[41] Kielbassa AM, Munz I, Bruggmoser G, Schulte-Mönting J (2002). Effect of demineralization and remineralization on microhardness of irradiated dentin. J Clin Dent.

[42] Scribante A, Farahani MRD, Marion G, et al. Biomimetic effect of nanohydroxyapatite in demineralized enamel before orthodontic bonding of brackets and attachments: visual, adhesion strength, and hardness in vitro tests. BioMed Res Int. 2020;2020:6747498. 10.1155/2020/6747498 PMC7013302 32090106

[43] Qiu W, Zhou Y, Li Z, Huang T, Xiao Y, Cheng L, et al. Application of antibiotics/antimicrobial agents on dental caries. BioMed Res Int. 2020;2020:5658212. 10.1155/2020/5658212 PMC7013294 32076608

[44] Honkala S, Runnel R, Saag M, Olak J, Nõmmela R, Russak S (2014). Effect of erythritol and xylitol on dental caries prevention in children. Caries Res.

[45] Liu BY, Lo ECM, Chu CH, Lin HC (2012). Randomized trial on fluorides and sealants for fissure caries prevention. J Dent Res.

[46] Kumar JV, Moss ME (2008). Fluorides in dental public health programs. Dent Clin North Am.

[47] Murakami C, Bönecker M, Corrêa MSNP, Mendes FM, Rodrigues CR (2009). Effect of fluoride varnish and gel on dental erosion in primary and permanent teeth. Arch Oral Biol.

[48] Alamoudi SA, Pani SC, Alomari M. The effect of the addition of tricalcium phosphate to 5% sodium fluoride varnishes on the microhardness of enamel of primary teeth. Int J Dent. 2013;2013:486358. 10.1155/2013/486358 PMC3679819 23781245

[49] Tuloglu N, Bayrak S, Tunc ES, Ozer F (2016). Effect of fluoride varnish with added casein phosphopeptide-amorphous calcium phosphate on the acid resistance of the primary enamel. BMC Oral Health..

[50] Oliveira MRC, Oliveira PHC, Oliveira LHC, Horliana ACRT, César PF, Moura SK (2019). Microhardness of bovine enamel after different fluoride application protocols. Dent Mater J.

[51] Ryu J, Ku SH, Lee H, Park CB (2010). Mussel-inspired polydopamine coating as a universal route to hydroxyapatite crystallization. Adv Funct Mater.

[52] Wang H, Lin C, Zhang X, Lin K, Wang X, Shen SG (2019). Mussel-inspired polydopamine coating: A general strategy to enhance osteogenic differentiation and osseointegration for diverse implants. ACS Appl Mater Interfaces.

[53] Wu M, Wang T, Wang Y, Wang H (2019). Ultrafast bone-like apatite formation on bioactive tricalcium silicate cement using mussel-inspired polydopamine. Ceram Int.

[54] Zhou Y-Z, Cao Y, Liu W (2012). ’Polydopamine-induced tooth remineralization’. ACS Appl Materilas Interfaces.

[55] Chu CH, Lo ECM (2008). Microhardness of dentine in primary teeth after topical fluoride applications. J Dent.

[56] Yeh ST, Wang HT, Liao HY, Su SL, Chang CC, Kao HC (2011). The roughness, microhardness, and surface analysis of nanocomposites after application of topical fluoride gels. Dent Mater.

[57] Vieira AEM, Delbem ACB, Sassaki KT, Rodrigues E, Cury JA, Cunha RF (2005). Fluoride dose response in pH-cycling models using bovine enamel. Caries Res.

[58] Arnaud TMS, de Barros NB, Diniz FB (2010). Chitosan effect on dental enamel de-remineralization: an in vitro evaluation. J Dent.

[59] Hicks J, Garcia-Godoy F, Flaitz C (2003). Biological factors in dental caries: role of saliva and dental plaque in the dynamic process of demineralization and remineralization (part 1). J Clin Pediatr Dent.

